# Prospective observational study on assessing the hemodynamic relevance of patent ductus arteriosus with frequency domain near-infrared spectroscopy

**DOI:** 10.1186/s12887-018-1054-6

**Published:** 2018-02-16

**Authors:** Christoph E. Schwarz, Antonio Preusche, Martin Wolf, Christian F. Poets, Axel R. Franz

**Affiliations:** 1 Department of Neonatology, University Children’s Hospital, Calwerstr. 7, 72076 Tuebingen, Germany; 2 Department of Neonatology, University Children’s Hospital, Tuebingen, Germany; 3 Department of Neonatology, University Hospital Zurich, Zurich, Switzerland; 4 Department of Neonatology, University Children’s Hospital, Tuebingen, Germany; 5 Department of Neonatology, University Children’s Hospital, Tuebingen, Germany; 6 Center for Pediatric Clinical Studies, University Children’s Hospital, Tuebingen, Germany

**Keywords:** Doppler-ultrasound, Cerebral oxygenation, Tissue oxygenation, Near-infrared spectroscopy, Echocardiography, Patent ductus arteriosus, Preterm infants

## Abstract

**Background:**

What constitutes a hemodynamically relevant patent ductus arteriosus (hrPDA) in preterm infants is unclear. Different clinical and echocardiographic parameters are used, but a gold standard definition is lacking.

Our objective was to evaluate associations between regional cerebral tissue oxygen saturation (rcStO_2_), fraction of tissue oxygen extraction (rcFtO_2_E) measured by frequency domain near-infrared spectroscopy (fd-NIRS) and their correlation to echocardiographic, Doppler-ultrasound, and clinical parameters in preterm infants with and without a hrPDA.

**Methods:**

In this prospective observational study, 22 infants < 1500 g (mean [± SD]: gestational age 28.6 [±1.8] weeks, birth weight 1076 [±284] g, median (interquartile range) postnatal age at measurement 7.6 (4.6–12.9) d) with a clinical suspicion of hrPDA were analysed.

Twelve infants had left-to-right shunt through PDA, and in 6 of these the PDA was classified as hrPDA based on pre-defined clinical and echocardiographic criteria. fd-NIRS, echocardiographic and Doppler-ultrasound examinations were performed. After identification of blood hemoglobin (Hb) as confounding factor, rcStO_2_ and rcFtO_2_E were corrected for this effect.

**Results:**

Overall mean ± standard deviation (normalised to a median Hb of 13.8 mg/dl) was 57 ±5% for rcStO_2_ and 0.39 ±0.05 for rcFtO_2_E. Comparing no-hrPDA with hrPDA infants, there were no significant differences in mean rcStO_2_ (58 ±5% vs. 54 ±5%; *p* = .102), but in mean rcFtO_2_E (0.38 ±0.05 vs. 0.43 ±0.05; *p* = .038). Echocardiographic parameter and Doppler indices did not correlate with cerebral oxygenation.

**Conclusion:**

Oxygen transport capacity of the blood may confound NIRS data interpretation. Cerebral oxygenation determined by fd-NIRS provided additional information for PDA treatment decisions not offered by routine investigations. Whether indicating PDA therapy based on echocardiography complemented by data on cerebral oxygenation results in better outcomes should be investigated in future studies.

## Background

Screening for, and therapy of, patent ductus arteriosus (PDA) in preterm infants is associated with decreased mortality and morbidity [1]. However, there is little evidence as to which parameters define a PDA that requires treatment, i.e. is hemodynamically relevant (hrPDA). Zonnenberg and de Waal summarized which echocardiographic and Doppler-ultrasound measurements, besides clinical parameters, have been used to evaluate the magnitude and clinical relevance of left-to-right shunting through a PDA [2]. Scoring Systems like the one suggested by McNamara and Sehgal include clinical and echocardiographic criteria to define hrPDA [3]. The echocardiographic part of this staging is useful to predict neonatal morbidity and may serve as a guide to clinical decision-making [4]. Resistive index (RI) in the anterior cerebral artery (ACA) was shown to be significantly higher in infants with hrPDA [5].

With frequency domain near-infrared spectroscopy (fd-NIRS) regional cerebral tissue oxygen saturation (rcStO_2_) and (together with pulse oximetry data) tissue oxygen extraction (rcFtO_2_E) may be monitored noninvasively. In a recent observational study using continuous wave (cw) NIRS, significant differences in cerebral oxygenation over time were observed in infants born below 32 weeks gestation within their first 6 postnatal days [6]. Such an effect, however, was not identified in other recent studies using this technique [7, 8].

To inform future studies and clinical guidelines on PDA treatment, this study aimed to evaluate the absolute cerebral oxygenation of preterm infants besides echocardiographic and Doppler-ultrasound parameters, which are frequently determined to assess the need for PDA treatment.

## Methods

This prospective observational cohort study was approved by the institutional research ethics committee and written informed parental consent obtained. Inclusion criteria were gestational age (GA) <37 weeks, birth weight (bw) ≤1500 g and clinical suspicion of hrPDA such as cardiac murmur, bounding pulses, ventilator dependency, metabolic acidosis and increased oxygen requirements. Syndromal anomalies and congenital heart defects except persisting foramen ovale or atrial septal defect were exclusion criteria. Recruitment took place from June 2012 to May 2013 and September 2015 to March 2016 at the tertiary level neonatal intensive care unit of the university children’s hospital, Tuebingen, Germany.

### Fd-NIRS-measurements

In infants with clinical indication for echocardiography based on a clinical suspicion of PDA, fd-NIRS was measured at the time of echocardiography. The reflectance mode multi-distance fd-NIRS device OxiplexTS (ISS Inc. Champaign, Illinois, U.S.A.) measures mean intensity (DC), modulation amplitude (AC) and phase shift (PH) at 4 distances and 2 wavelengths and determines absolute values of cerebral oxy-, deoxy-, total hemoglobin concentration and rcStO_2_. The optode was placed, as previously described [9], in a temporo-parietal position half-way between tragus and sagittal suture. The optode was held by the examiner for 5 consecutive measurements of 1 min each at either side of the head; it was repositioned between measurement runs [9]. The tissue water content was assumed to be 90% [10]. To exclude effects of tissue inhomogeneity, a quality criteria algorithm was used with r^2^ >0.8 in the linear regression analyses for AC, DC and PH over the four emitter-optode distances as surrogate according to Arri et al. [11]. We decided to use this cut-off because higher cut-offs for r^2^ were unable to increase precision significantly but led to loss of information. To overcome inaccuracy of single measurements [12] we used the mean of up to 10 measurements over 1 min each. Recording frequency was 1 Hz. Intra-observer repeatability was evaluated post-hoc by within-subject SD (SD_W_) estimated as square root of the residual mean square by oneway-ANOVA with subject as factor according to Hyttel-Sorensen et al. [13]. The SD_W_ was ±7% for rcStO_2_ and ±0.08 for rcFtO_2_E.

### Echocardiographic and Doppler-ultrasound measurements

The Parameters assessed were LA/Ao-ratio [14], diameter of the PDA at its narrowest part [15], the left-ventricular-preejection-period-to-ejection-time-ratio, calculated by including 3–4 cardiac cycles (LVPEP/LVET) [16], and the ratio of the velocity time integrals in the large vessels aorta and pulmonary artery (VTI_Ao/VTI_PA). The VTI_Ao was recorded from an apical-5-chamber-view, the VTI_PA in a parasternal short axis. We assumed that, in the absence of congenital heart defects, this ratio correlates with the ductal left-to-right shunt. After visualisation of the whole course of the PDA, its diameter was measured at its narrowest part (identified via colour-Doppler in the high left-sided parasternal “ductal” view and the suprasternal view) and measured in B-Mode to avoid the influence of gain-settings on the PDA-width. RI in celiac artery (CA) [17] and ACA [18] were measured as well. Based on our previous analyses on repeatability of echocardiographic parameters [19], mean values of repeated measurements were assessed whenever possible (i.e., in 18/22 infants, in 4 patients only single measurements were available).

All measurements were performed with a Toshiba “Aplio” using a 6.5 MHz phased array transducer (Toshiba Medical Systems, Otawara-shi, Tochigi-ken, Japan) or a Zonare ZS3 using C10–3 curved-phased array transducer (Zonare Medical Systems, Inc., Mountain View, CA, U.S.A.). For calculation of velocity time integrals, the former device enables automatic detection while the latter device requires manual circumscription of the Doppler wave form.

### Cardiorespiratory monitoring and laboratory investigations

During measurements, heart rate (HR) and peripheral arterial oxygen saturation (SpO_2_) were monitored in all patients via pulse oximetry (Radical 7; Masimo Corporation, Irvine, CA, U.S.A.) and the SpO_2_ was targeted at 90–95% if on supplemental oxygen and at > 89% if no supplemental oxygen was used. Averaging and recording intervals were 2 s each. As for fd-NIRS measurements, we calculated means for each 1-min measurement of these parameters. Blood hemoglobin concentration (Hb in g/dL) from clinically indicated whole blood counts was extracted from patients’ charts if determined within ±12 h of NIRS-measurements or by linear interpolation of the two adjacent Hb concentrations.

### Definition of hrPDA

According to the clinical standard in use in the unit, hrPDA was defined if a left-to-right shunt through a PDA was confirmed by echocardiography and at least 3 of the following 6 criteria were met:

1. LA/Ao-ratio >1.5
2. PDA diameter ≥1.5 mm/kg bodyweight
3. Need of respiratory support (mechanical Ventilation or continuous positive airway pressure with supplemental oxygen)
4. Reverse or zero end diastolic flow in ACA (=RI_ACA ≥ 1)
5. Reverse or zero end diastolic flow in CA (=RI_ CA ≥ 1)
6. LVPEP/LVET <0.32

Infants with left-to-right shunt through a PDA meeting less than 3 of the above listed criteria were classified as hiPDA (hemodynamically irrelevant) and allocated, together with infants without a PDA, to the no-hrPDA group.

### Statistical analyses

RcStO_2_ was extracted and rcFtO_2_E calculated from rcStO_2_ and SpO_2_ data according to Naulaers et al. [20]. Results were screened for potential confounding factors (GA, BW, age at measurement (AAM), gender, Hb, small for gestational age (SGA)) by Spearman-Rho/Pearson correlation coefficient and Fisher’s exact test, as appropriate. If a statistically significant influence was identified on univariate analysis, rcStO_2_ and rcFtO_2_E were normalized for the median of this confounding factor.

Patients were stratified into 2 groups: no-hrPDA and hrPDA according to echocardiographic and clinical parameters (see definition of hrPDA). Demographic, neonatal and echocardiographic parameters were tested for significant group differences using t-, Mann-Whitney-U and Fisher’s exact test, oneway-ANOVA or Welch-Test, as appropriate.

RcStO_2_ and rcFtO_2_E were tested for statistically significant differences with oneway-ANOVA after confirmation of normal distribution (Shapiro-Wilk) and homogeneity of variances (Levene-statistics).

A *p*-value below.05 was set to indicate statistical significance. Data are presented as median and interquartile range (IQR) or mean and ± standard deviation (±SD) if not noted otherwise. Where appropriate, 95% confidence intervals (CI) were added. SPSS-Statistics (Version 24.0.0.0, IBM Corporation, Armonk, NY, U.S.A.) was used for statistical analyses.

## Results

### Demographic and neonatal parameters

Twenty-four infants were included in the study. One infant with a BW of 1550 g was included inadvertently due to a body weight at the time of measurement of 1465 g (hiPDA). Two infants were excluded: one infant of 23 weeks GA, BW 360 g, due to fulminant sepsis a few hours after inclusion leading to its demise (hrPDA), so that cerebral oxygenation and hemodynamics could have been corrupted, the other (GA 26 weeks, BW 745 g) due to a ventricular septal defect found during his study echocardiography (hiPDA). Demographic and clinical data of the 22 preterm infants analysed in this cohort are summarized in Table 1.

**Table 1:** Neonatal characteristics and influence factors

Neonatal variable	hrPDA *n* = 6	no-hrPDA *n* = 16	*p*
Gestational age (weeks)	28.5 (25.9–29.4)	28.8 (28.5–29.7)	0.33
Birth weight (g)	1110 (793–1128)	1090 (879–1346)	0.43
Small for gestational age	0	2	
Male	2	11	0.18
Age at measurement (d)	4.7 (4.6–7.2)	8.1 (4.5–21.7)	0.26
Weight at measurement (g)	1118 (788–1162)	1233 (1001–1675)	0.23
Mechanical Ventilation	2	2	
CPAP	4	9	
Fraction of inspired Oxygen	0.23	0.21	0.80
Hb (g/dl)	15.0 (13.9–15.2)	12.6 (11.2–14.6)	0.08
SpO_2_ (%)	94 (93–96)	95 (92–95)	0.51
HR (1/min)	163 (158–175)	161 (154–168)	0.33
meanBP (mmHg)	37 (35–40)	46 (41–49)	0.01
Inotropes	0	0	

Values are median (interquartile range) or n; CPAP: continuous positive airway pressure, Hb: hemoglobin concentration, SpO_2_: pulse oximetry measured arterial oxygen saturation, HR: heart rate, meanBP: mean blood pressure

Three infants in the hrPDA group had indomethacin within 24 h prior to measurements (0.1/0.2/0.4 mg/kg BW/day, respectively). Overall, 5 patients needed inotropes and 6 had medical therapy for PDA closure according to the clinical standard in use in the unit. No infant underwent surgical closure of PDA.

### Echocardiographic und Doppler-ultrasound measurements

A left-to-right shunt through PDA was identified by colour-Doppler-ultrasound in 12/22 measurements. PDA-diameter at the narrowest part could rarely be measured (*n* = 6) due to difficulties visualizing the PDA in its complete course in B-mode. PDA staging classified 6 PDAs as hrPDA and 6 as hiPDA. All 6 parameters used to define hrPDA were relevantly different between groups. Mean VTI_Ao/VTI_PA in hrPDA vs. no-hrPDA was 1.43 ±0.56 (95% CI 0.84, 2.02) vs. 0.93 ±0.24 (95% CI 0.80,1.06), *p* = .085 (Table 2).

**Table 2:** Echocardiography/Doppler-ultrasound results

Parameter	hrPDA *n* = 6	no-hrPDA *n* = 16	*p*
Left-to-right shunt PDA	6	6	
LA/Ao	1.6 (1.4–1.8)	1.3 (1.2–1.5)	.15
*PDA_diameter/kgBW*	*1.6 (1.4–2.1)*	*0.6 (.6–.6)*	*ns*
RI_ACA	.86 (.83–.89)	.77 (.75–.82)	.01
RI_CA	1 (.86–1)	.77 (.71–.82)	.04
LVPEP/LVET	.26 (.21–.28)	.31 (.28–.33)	.09
VTI_Ao/VTI_PA	1.5 (.97–1.9)	.9 (.84–1.0)	.08

Values are median (interquartile range) or n; separated in hemodynamically relevant (hr) Patent ductus arteriosus (PDA) and no-hrPDA. Left atrium (LA), Aortic root (Ao), diameter of PDA, measured at its most constricted point, per kilogram of bodyweight (BW), Resistive indices (RI) in anterior cerebral artery (ACA) and celiac artery (CA), Left ventricular (LV) pre-ejection period (PEP) and ejection time (ET), Velocity time integral (VTI) and pulmonary artery (PA), bold: The ratio of VTI_Ao to VTI_PA was the only parameter not used in the definition of hrPDA, *italic*: PDA diameter/kgBW could rarely be measured (n = 6; 4 hrPDA and 2 no-hrPDA), statistical test: oneway-ANOVA or Welch-test, as appropriate

### NIRS and confounding factors

The oxygen transport capacity of the blood represented by Hb concentration was identified as a confounding factor on rcStO_2_ and rcFtO_2_E by Spearman-Rho (*p* = .022 and .004, respectively). Pearson correlation showed a significant influence as well (*p* = .023 and .003, respectively), supporting a linear relationship. We thus normalized rcStO_2_ and rcFtO_2_E for Hb using the linear regression equation:

$$ {\mathrm{normalized}\ \mathrm{rcStO}}_2=1,13{12}^{\ast}\left(\mathrm{medianHb}-\mathrm{patientHb}\right)+{\mathrm{measured}\ \mathrm{rcStO}}_2;{\mathrm{r}}^2=0.2338 $$

and

$$ {\mathrm{normalized}\ \mathrm{rcFtO}}_2\mathrm{E}=-0.01{53}^{\ast}\left(\mathrm{medianHb}-\mathrm{patientHb}\right)+{\mathrm{measured}\ \mathrm{rcFtO}}_2\mathrm{E};{\mathrm{r}}^2=0.3669 $$

After normalization for a median Hb value of 13.8 mg/dl, the overall mean rcStO_2_ was 57 ±5% (95% CI: 55%, 60%) and mean rcFtO_2_E 0.40 ± 0.05 (95% CI 0.37, 0.41), respectively. Mean RcStO_2_ of hrPDA vs. no-hrPDA infants was 54 ±5% (95% CI 49%, 59%) vs. 58 ±5% (95% CI 56%, 61%; *p* = .102). However, rcFtO_2_E mean was 0.43 ±0.05 (95% CI 0.38, 0.48) vs. 0.38 ±0.05 (95% CI 0.35, 0.40; *p* = .038) (Table 3).

**Table 3:** Cerebral Tissue Oximetry results

Parameter	hrPDA *n* = 6	no-hrPDA *n* = 16	*p*
rcStO_2_ (%)	54 (±5)	58 (±5)	.102
rcFtO_2_E	.43 (±.05)	.38 (±.05)	.038

Values are mean (±SD), test for significance: oneway-ANOVA, regional cerebral (rc) tissue oxygen saturation (StO_2_) and fraction of tissue oxygen extraction (FtO_2_E) normalized for median hemoglobin

None of the echocardiographic or Doppler-ultrasound parameters correlated significantly with Hb-normalized rcStO_2_ or Hb-normalized rcFtO_2_E.

## Discussion

Using fd-NIRS and normalization for Hb, we found a significant difference in rcFtO_2_E, but not in rcStO_2_, between infants with vs. without hrPDA in our cohort. As neither Hb-normalized rcStO_2_ nor rcFtO_2_E correlated with echocardiographic or Doppler-ultrasound parameters, fd-NIRS parameters of cerebral oxygenation may offer additional information and may help to better inform treatment decisions.

In general, a good diagnostic parameter can easily and quickly be determined, has high sensitivity and specificity and is not incriminating to measure in preterm infants. NIRS is a non-invasive technique, and lower cerebral oxygenation within the first 2 postnatal weeks has been associated with poorer neurodevelopmental long term outcome of preterm infants [21]. Suboptimal cerebral oxygenation due to a hrPDA may negatively influence brain growth at term-equivalent age [22] and, at least in animal models, rcStO_2_ values below 40% are identified as a risk factor for an impaired neurodevelopment [23].

Data on PDA-related changes in cerebral oxygenation are still controversial, although an impact on cerebral oxygenation has been identified in several studies. In a recent prospective observational trial involving 380 infants of <32 weeks gestation studied during their first 6 days postnatal days, Dix et al. found significant differences in cerebral oxygenation over time [6]. In line with these data, rcFtO_2_E showed a statistically significant difference between hr- and non-hrPDA, whereas rcStO_2_ did not, but this latter observation may be due limited power due to our small sample size. Another explanation might be an effect of (small) changes in arterial oxygen saturation during measurements, which are not addressed by rcStO_2_. Chock et al. studied 47 infants with GA below 29 weeks (21 echocardiographically stratified as hrPDA) and found no significant relationship to rcStO_2_ [7]. In line with this, others reported data on 20 hrPDA compared with 29 non-hrPDA preterm infants (median GA 27.6 weeks, median BW 980 g) and found no significant effect on rcStO_2_ or rcFtO_2_E [8]. Older observational trials found a pronounced impact of hrPDA on cerebral oxygenation in infants scheduled for surgical closure [24–28]. In the present cohort, no infants underwent surgical ligation due to a very restrictive policy on surgical PDA closure in our unit.

Contrary to previous studies using cw-NIRS devices, fd-NIRS provides absolute instead of trend values for rcStO_2_ under the assumption of a homogenous semi-infinite tissue. Another advantage of this technique is the opportunity of extracting unprocessed optical raw data, enabling the introduction of a quality pre-analytic algorithm for using high quality data for analysis, thereby improving repeatability. A disadvantage is that this instrument is less practical for continuous bedside trend monitoring due to the use of fragile glass fibre cables as well as its non-disposable probe.

Overall mean values for rcStO_2_ were within the (lower) normal range [29], taking into account that this “normal range” was established for the first 72 h of life, while our cohort consisted of older preterm infants. Both, device and sensor, but also the clinical suspicion of hrPDA and/or the higher postnatal age may have contributed to these slightly lower values [30].

A variety of confounding factors impacting on cerebral oxygenation as measured with NIRS are known. Growth restriction, gender and gestational age have already been identified [6, 31, 32]. We were unable to reproduce these findings, possibly due to our low sample size. Only Hb concentration was identified as a confounder so that rcStO_2_ and rcFtO_2_E were normalized for median Hb before further analysis. This should be addressed in future investigations. The higher rcFtO_2_E observed with hrPDA supports previous suggestions to maintain higher Hb concentrations in infants with hrPDA as part of conservative management [25]. Whether this might have an effect on long term outcome has not been studied yet.

We found no significant correlation between echocardiographic or Doppler-ultrasound parameters and cerebral oxygenation. A large number of different echocardiographic and Doppler-ultrasound parameters are used to quantify left-to-right shunting through, and hemodynamic relevance of, a PDA (summarised in [2]). This is reflected in NIRS-studies on hrPDA and its effect on cerebral oxygenation by relevant differences in hrPDA definition [7, 8, 33]. This substantially complicates comparisons between studies on hrPDA. The cut-offs used here reflect local preferences and the number of parameters tested was limited to reduce examination time and hence study-driven burden on infants.

To our knowledge only Dix et al. identified a significant relation between an echocardiographic parameter (ductal diameter) and rcStO_2_ [6]. We were only rarely able to measure PDA diameter due to our strict definition of the most constricted point at the whole course of the PDA in B-mode. In line with our results Dix et al. found no relation between LA/Ao and cerebral oxygenation.

Before embarking on this study, we assumed that the VTI_Ao/VTI_PA-ratio might be another easily determined parameter suitable for quantifying ductal left-to-right shunt under the assumption of a “normal” ratio of Ao to PA valve-area. It is also important to have in mind that it could be corrupted by inter-atrial shunting, which is commonly observed in VLBW infants (and also in 20 of the infants studied here). However, this limitation also applies to LA/Ao ratio, the most commonly used parameter. VTI_Ao/VTI_PA-ratio has a relatively low repeatability and could be corrupted by ductal jet, although it is supposed to allow discrimination between hrPDA and hiPDA [19].

Limitations of this study are its small sample size and that extremely low gestational age infants, who are most likely to develop hrPDA, were under-represented. We have been reluctant to subject the more immature and hence more vulnerable infants to the additional study-related burden.

By contrast, it is a strength of our study that fd-NIRS was used, truly generating absolute values for rcStO_2_ and rcFtO_2_E and that a rigorous quality assessment of the raw data preceded further analyses. Furthermore, we identified that Hb was an important confounder of cerebral oxygenation in this context and therefore assessed Hb-normalized values of NIRS parameters.

Only 5 patients overall were ever treated for PDA – this reflects the very restrictive approach to PDA treatment present in our unit - others may be more proactive [6].

## Conclusions

Oxygen transport capacity of the blood may be taken into account for NIRS data interpretation. In our cohort we found a statistically significant difference in absolute cerebral oxygenation between clinically and echocardiographically defined hrPDA compared to no-hrPDA infants. Furthermore, our data suggest that parameters of cerebral oxygenation might offer additional information for the clinical decision whether or not to treat a PDA in an individual infant. Whether indicating PDA therapy based on echocardiography complemented by data on cerebral oxygenation results in better outcomes should be investigated in future studies.

## Abbreviations

AAM: Age at measurement
AC: Modulation amplitude
ACA: Anterior cerebral artery
BW: Birthweight
CA: Celiac artery
CI: Confidence interval
cw: Continuous wave
DC: Mean intensity
fd: Frequency domain
GA: Gestational age
Hb: Hemoglobin concentration
hiPDA: Hemodynamically irrelevant PDA
hrPDA: Hemodynamically relevant PDA
IQR: Interquartile range
LA/Ao: Left-atrium-to-aortic-root-ratio
LVPEP/LVET: Left-ventricular-preejection-period-to-ejection-time-ratio
NIRS: Near-infrared spectroscopy
PDA: Patent ductus arteriosus
PH: Phase shift
rcFtO_2_E: Regional cerebral fraction of tissue oxygen extraction
rcStO_2_: Regional cerebral tissue oxygen saturation
RI: Resistive index
SD: Standard deviation
SGA: Small for gestational age
SpO_2_: Pulse oximetry measured arterial oxygen saturation
VTI_Ao: Velocity time integral ascending Aorta
VTI_PA: Velocity time integral pulmonary artery
WAM: Body weight at measurement

## References

[1] Rozé JCCG, Marchand-Martin L, Gournay V, Durrmeyer X, Durox M, Storme L, Porcher R, Ancel PY, Hemodynamic EPIPAGE 2 Study Group (2015). Association between early screening for patent ductus arteriosus and in-hospital mortality among extremely preterm infants. JAMA.

[2] Zonnenberg I, de Waal K (2012). The definition of a haemodynamic significant duct in randomized controlled trials: a systematic literature review. Acta Paediatr.

[3] McNamara PJ, Sehgal A (2007). Towards rational management of the patent ductus arteriosus: the need for disease staging. Arch Dis Child Fetal Neonatal Edition.

[4] Schena FFG, Cappelleri A, Picciolli I, Mayer A, Mosca F, Fumagalli M (2015). Association between hemodynamically significant patent ductus arteriosus and bronchopulmonary dysplasia. J Pediatr.

[5] Ecury-Goossen GM, Raets MM, Camfferman FA, Vos RH, van Rosmalen J, Reiss IK, Govaert P, Dudink J (2016). Resistive indices of cerebral arteries in very preterm infants: values throughout stay in the neonatal intensive care unit and impact of patent ductus arteriosus. Pediatr Radiol.

[6] Dix L, Molenschot M, Breur J, de Vries W, Vijlbrief D, Groenendaal F, van Bel F, Lemmers P (2016). Cerebral oxygenation and echocardiographic parameters in preterm neonates with a patent ductus arteriosus: an observational study. Arch Dis Child Fetal Neonatal Ed.

[7] Chock VY, Rose LA, Mante JV, Punn R (2016). Near-infrared spectroscopy for detection of a significant patent ductus arteriosus. Pediatr Res.

[8] van der Laan ME, Roofthooft MT, Fries MW, Berger RM, Schat TE, van Zoonen AG, Tanis JC, Bos AF, Kooi EM (2016). A hemodynamically significant patent ductus arteriosus does not affect cerebral or renal tissue oxygenation in preterm infants. Neonatology.

[9] Demel A, Feilke K, Schoning M, Wolf M, Poets CF, Franz AR (2015). Healthy term and moderately preterm infants have similar cerebral oxygen saturation and cerebral blood flow volumes during early post-natal transition. Acta Paediatr.

[10] Demel A, Wolf M, Poets CF, Franz AR (2014). Effect of different assumptions for brain water content on absolute measures of cerebral oxygenation determined by frequency-domain near-infrared spectroscopy in preterm infants: an observational study. BMC Pediatr.

[11] Arri SJ, Muehlemann T, Biallas M, Bucher HU, Wolf M (2011). Precision of cerebral oxygenation and hemoglobin concentration measurements in neonates measured by near-infrared spectroscopy. J Biomed Opt.

[12] Sorensen LC, Greisen G (2006). Precision of measurement of cerebral tissue oxygenation index using near-infrared spectroscopy in preterm neonates. J Biomed Opt.

[13] Hyttel-Sorensen S, Hessel TW, Greisen G (2014). Peripheral tissue oximetry: comparing three commercial near-infrared spectroscopy oximeters on the forearm. J Clin Monit Comput.

[14] Green TP, Thompson TR, Johnson DE, Lock JE (1983). Furosemide promotes patent ductus arteriosus in premature infants with the respiratory-distress syndrome. N Engl J Med.

[15] Kluckow M, Evans N (1995). Early echocardiographic prediction of symptomatic patent ductus arteriosus in preterm infants undergoing mechanical ventilation. J Pediatr.

[16] Robel-Tillig E, Knupfer M, Pulzer F, Vogtmann C (2002). Dopplersonographic findings in neonates with significant persistent ductus arteriosus. Z Geburtshilfe Neonatol.

[17] Shimada S, Kasai T, Konishi M, Fujiwara T (1994). Effects of patent ductus arteriosus on left ventricular output and organ blood flows in preterm infants with respiratory distress syndrome treated with surfactant. J Pediatr.

[18] Martin CG, Snider AR, Katz SM, Peabody JL, Brady JP (1982). Abnormal cerebral blood flow patterns in preterm infants with a large patent ductus arteriosus. J Pediatr.

[19] Schwarz CE, Preusche A, Baden W, Poets CF, Franz AR (2016). Repeatability of echocardiographic parameters to evaluate the hemodynamic relevance of patent ductus arteriosus in preterm infants: a prospective observational study. BMC Pediatr.

[20] Naulaers G, Meyns B, Miserez M, Leunens V, Van Huffel S, Casaer P, Weindling M, Devlieger H (2007). Use of tissue oxygenation index and fractional tissue oxygen extraction as non-invasive parameters for cerebral oxygenation. A validation study in piglets. Neonatology.

[21] Verhagen EA, Van Braeckel KN, van der Veere CN, Groen H, Dijk PH, Hulzebos CV, Bos AF (2015). Cerebral oxygenation is associated with neurodevelopmental outcome of preterm children at age 2 to 3 years. Dev Med Child Neurol.

[22] Lemmers PM, Benders MJ, D'Ascenzo R, Zethof J, Alderliesten T, Kersbergen KJ, Isgum I, de Vries LS, Groenendaal F, van Bel F (2016). Patent ductus arteriosus and brain volume. Pediatrics.

[23] Hou X, Ding H, Teng Y, Zhou C, Tang X, Li S, Ding H (2007). Research on the relationship between brain anoxia at different regional oxygen saturations and brain damage using near-infrared spectroscopy. Physiol Meas.

[24] Lemmers PM, Molenschot MC, Evens J, Toet MC, van Bel F (2010). Is cerebral oxygen supply compromised in preterm infants undergoing surgical closure for patent ductus arteriosus?. Arch Dis Child Fetal Neonatal Ed.

[25] Chock VY, Ramamoorthy C, Van Meurs KP (2011). Cerebral oxygenation during different treatment strategies for a patent ductus arteriosus. Neonatology.

[26] Zaramella P, Freato F, Quaresima V, Ferrari M, Bartocci M, Rubino M, Falcon E, Chiandetti L (2006). Surgical closure of patent ductus arteriosus reduces the cerebral tissue oxygenation index in preterm infants: a near-infrared spectroscopy and Doppler study. Pediatr Int.

[27] Huning BM, Asfour B, Konig S, Hess N, Roll C (2008). Cerebral blood volume changes during closure by surgery of patent ductus arteriosus. Arch Dis Child Fetal Neonatal Ed.

[28] Vanderhaegen J, De Smet D, Meyns B, Van De Velde M, Van Huffel S, Naulaers G (2008). Surgical closure of the patent ductus arteriosus and its effect on the cerebral tissue oxygenation. Acta Paediatr.

[29] Alderliesten T, Dix L, Baerts W, Caicedo A, van Huffel S, Naulaers G, Groenendaal F, van Bel F, Lemmers P (2016). Reference values of regional cerebral oxygen saturation during the first 3 days of life in preterm neonates. Pediatr Res.

[30] Kleiser S, Hyttel-Sorensen S, Greisen G, Wolf M (2016). Comparison of near-infrared oximeters in a liquid optical phantom with varying Intralipid and blood content. Adv Exp Med Biol.

[31] Cohen E, Baerts W, Alderliesten T, Derks J, Lemmers P, van Bel F (2016). Growth restriction and gender influence cerebral oxygenation in preterm neonates. Arch Dis Child Fetal Neonatal Ed.

[32] Cohen E, Dix L, Baerts W, Alderliesten T, Lemmers P, van Bel F (2016). Reduction in cerebral oxygenation due to patent ductus arteriosus is pronounced in small-for-gestational-age neonates. Neonatology.

[33] Petrova A, Bhatt M, Mehta R (2011). Regional tissue oxygenation in preterm born infants in association with echocardiographically significant patent ductus arteriosus. J Perinatol.

